# Silencing SOX2 Induced Mesenchymal-Epithelial Transition and Its Expression Predicts Liver and Lymph Node Metastasis of CRC Patients

**DOI:** 10.1371/journal.pone.0041335

**Published:** 2012-08-17

**Authors:** Xu Han, Xuefeng Fang, Xiaoyan Lou, Dasong Hua, Wenchao Ding, Gregory Foltz, Leroy Hood, Ying Yuan, Biaoyang Lin

**Affiliations:** 1 Systems Biology Division, Zhejiang–California International Nanosystems Institute, Zhejiang University, Hangzhou, Zhejiang, China; 2 Swedish Neuroscience Institute, Swedish Medical Center, Seattle, Washington, United States of America; 3 The Institute for Systems Biology, Seattle, Washington, United States of America; 4 Department of Medical Oncology, The 2nd Hospital of Zhejiang University Medical College, Hangzhou, Zhejiang, China; 5 Department of Urology, University of Washington, Seattle, Washington, United States of America; University of Colorado Boulder, United States of America

## Abstract

SOX2 is an important stem cell marker and plays important roles in development and carcinogenesis. However, the role of SOX2 in Epithelial-Mesenchymal Transition has not been investigated. We demonstrated, for the first time, that SOX2 is involved in the Epithelial-Mesenchymal Transition (EMT) process as knock downof SOX2 in colorectal cancer (CRC) SW620 cells induced a Mesenchymal-Epithelial Transition (MET) process with recognized changes in the expression of key genes involved in the EMT process including E-cadherin and vimentin. In addition, we provided a link between SOX2 activity and the WNT pathway by showing that knock down of SOX2 reduced the WNT pathway activity in colorectal cancer (CRC) cells. We further demonstrated that SOX2 is involved in cell migration and invasion *in vitro* and in metastasis *in vivo* for CRC cells, and that the process might be mediated through the MMP2 activity. Finally, an IHC analysis of 44 cases of colorectal cancer patients suggested that SOX2 is a prognosis marker for metastasis of colorectal cancers.

## Introduction

The epithelial to mesenchymal transition (EMT) is well-coordinated process during embryonic development as well as progression of cancers including colorectal cancers [1]–[4]. Epithelial cells gain polarity and motility during EMT, which are necessary for tumor invasion and metastasis in different types of epithelial carcinomas [3], [5]. For example, colorectal cancer (CRC) cells at the invasive front usually acquire mesenchymal properties including highly migratory, poorly differentiated, hyperproliferative, and loss of cell-cell contact–mediated growth inhibition [4].

*SOX2* is one of the key members of the SOX family gene and plays critical role in embryonic stem cells [6] and in induced pluripotent stem cells [7]–[10]. It is also involved in invasion and metastasis of pancreatic carcinoma [11], and in carcinogenesis of gastric [12], breast [13], pancreatic cancers [11], and osteosarcomas [14] and glioma [15], [16]. Furthermore, *SOX2* also maintains self-renewal of cancer stem cells [14] or is activated in cancer stem cells [17].

An intriguing question to ask is whether cancer cells in epithelial-to-mesenchymal transition and tumor-propagating–cancer stem cells are distinct, overlapping or same populations [18]. Mani et al. reported that induction of EMT in human mammary epithelial cells (HMLEs) resulted in the gain of epithelial stem cell properties in HMLEs [19]. In this work, we asked the question whether the key stem cell gene SOX2 plays a role in the EMT process. We used colorectal cancer as a.model to address the question. As a result, we demonstrated that SOX2 knock down in colorectal cell (CRC) SW620 induced a Mesenchymal-Epithelial Transition (MET) process, with characteristic morphological changes from spindle and fibroblastoid shape to cobblestone-like cell shape, and with associated changes in expression of key genes involved in the MET process including E-cadherin and vimentin. In addition, MMP2 activity and the WNT pathway activity were decreased significantly in the SOX2 knock down colorectal cells. We further showed that knocking down SOX2 could inhibit cell mobility and invasion *in vitro* and suppress metastasis *in vivo for* CRC cells. Finally, we showed that elevated expression of SOX2 is significantly correlated with metastases in CRCs. Our manuscript describes, for the first time, a novel role of SOX2 in regulating the EMT process in cancers.

## Materials and Methods

### Cell culture

The human colorectal cell line SW620 was a kindly gift from The Second Affiliated Hospital, Zhejiang University School of Medicine. The stable transfected cells, SW620^mock^ and SW620^shRNA-SOX2^, were cultured in RMPI-1640 medium with 10% fetal bovine serum.

### Immunofluorescence cell staining

Immunofluorescence cell staining was performed using the following primary antibodies: Rabbit anti-SOX2 (Epitomics), 1∶250; mouse anti-Vimentin (Boster), 1∶100; mouse anti-E-cadherin (Abcam), 1∶100; and rabbit anti-beta-catenin (Epitomics), 1∶250. Cells were seeded on the cover slips and incubated for 24 hours at room temperature, and then fixed with formalin for 20 min, washed with PBS and blocked with PBS containing 1% of BSA and 0.25% Triton X-100. Slides were incubated with primary antibody overnight at 4°C, washed with PBS, and then incubated with secondary antibody conjugated with FITC (green) or Cy3 (red) (Millipore) for 1 hour. After washing, cover slips were attached to glass slides. Cells were imaged using a confocal microscope.

### Western Blot Analysis

For whole-cell extract, cells were grown to 70% confluency, then washed with cold PBS buffer, and lysed on ice for 30 min in 200 ul RIPA buffer. Cells lysates were cleared by centrifuging at 14,000 rpm for 15 minutes. For nuclear extract, cells were lysed using NE-PER Nuclear and Cytoplasmic Extraction Reagents (Thermo SCIENTIFIC). Protein concentrations were estimated using Pierce BCA protein Assay Kit (Thermo SCIENTIFIC). 30 µg of proteins was denatured at 95° with loading buffer for 5 min and separated by electrophoresis in 12% SDS-PAGE gels for low molecular weight proteins. Proteins were transferred onto a PVDF membrane, blocked 1 hour in 5% slim milk in TBST. Primary antibodies were diluted to incubate with PVDF membrane overnight at 4° [anti-SOX2 (2683-1 Epitomics), 1∶5000; anti-Vimentin (BM0135, Boster), 1∶200; anti-E-cadherin (ab1416, Abcam), 1∶1000; anti-GAPDH (ab9483, Abcam), 1∶5000; anti-beta-catenin (1247-1, Epitomics), 1∶5000; anti-Lamin B (sc-365962, Santa cruz), 1∶500; anti-alpha-tublin (2871-1, Epitomics), 1∶5000; anti-TCF/LEF1, 1∶1000 (2091-1, Epitomics)]. After washing, the membranes were incubated for 1 h at RT temperature with HRP linked secondary antibody [anti-rabbit (1∶5000), anti-mouse (1∶5000) (Abcam)], and signals were detected using ECL reagents (Thermo Scientific).

### RNA isolation and RT-PCR

Total RNA was extracted after 24 hours postseeding with Trizol. 2 ug RNA for each analyzed sample was reverse transcribed in a final volume of 25 µl using M-MLV Reverse Transcriptase (Promega). Quantitative real-time PCR reaction was performed in a final volume of 20 µl containing 1 µl of cDNA (1∶10 dilution) and 400 nM of primers (All primers were listed in supplementary table S1) and SybrGreen PCR premix (Takara), and run in a Biorad Cycler. All samples were amplified in triplicate using the following cycle scheme: 95°C for 2 min, 35 cycles of 95°C for 15 s, 60°C for 30 sec and 72°C for 20 sec. Final mRNA levels were normalized to GAPDH levels. All results are plotted as columns (mean) and bars (SD).

### Cell migration and invasion assays

Cell migration assay was performed as described using Transwells (24-well Insent; 8 mM pore size polycarbonate membrane) obtained from Corning. Cells (5×10^5^) in 0.5 ml serum-free medium were placed in the upper chamber, whereas the lower chamber was loaded with 0.8 ml medium containing 10% FBS. The total number of cells that migrated into the lower chamber was counted after 60 h of incubation at 37°C with 5% CO_2_. Six visual fields were chosen for counting. The transwell cell invasion assay was performed using transwells that were pre-loaded with a layer of Matri-gel (Sigma-Aldrich) on the upper surface. The rest experiment procedure is same with cell migration assay.

### *In vivo* studies

All animal experiments were approved by governmental authorities. Six-week old female nude mice were each injected via the tail vein with SW620^shRNA-SOX2^ or SW620^mock^ (5×10^6^). Mice were sacrificed 30 days after the tail vein injection, and lung and liver were embedded in paraffin and sliced for HE staining to examine for metastasis.

### Gelatin zymography

Cells were plated at a density of 10^6^ in 6-well plates. After 16 hours, cells were washed with PBS and incubated in 300 ml of serum-free medium for 24 hours. 55 µl aliquots of conditioned medium were diluted in sample buffer (5% SDS), 20% glycerol in 0.4M Tris pH 6.8 containing 0.02% bromophenol blue without 2-mercaptoethanol and were loaded for zymography on a 10% polyacrylamide gel containing 1 mg/ml gelatin. Afterwards, gels were washed for 30 minutes in 2.5% Triton X-100 and incubated for 24 hours at 37°C in 50 mM Tris, pH 7.5, 10 mM CaCl_2_, 0.02% NaAzide. The buffer was decanted and the gels stained with 0.5% Coomassie blue G in 30% methanol and 10% acetic acid for 10 minutes at room temperature on a rotary shaker. Stain was washed out with water until clear bands were seen. Areas where proteolytic activity degraded the gelatin manifested as absence of staining.

### Immunohischemistry

Paraffin-embedded tumors samples were obtained from 44 colorectal cancer patients with approval from the institutional ethics committee of the 2nd hospital of Zhejiang University Medical College. Immunohistochemical (IHC) analyses were performed on 3-µm, formalin-fixed and paraffin-embedded sections, after microwave-assisted antigen retrieval in 0.1 mol/L of citrate buffer (pH 6.0) and subsequent incubation with 3% hydrogen peroxide. Primary antibodies for SOX2 were diluted at 1∶250 (Epitomics) and secondary antibody was used at 1∶200 dilution for IHC. Stained sections were photographed using a fluorescence microscope.

### Statistical analysis

The results are reported as mean ± SD. Statistical analysis was performed using Student's t-test or Chi-square test as appropriate. P<0.05 was considered to be statistically significant.

## Results

### Silencing SOX2 Promotes Mesenchymal-Epithelial Transition (MET) in Colorectal Cancer Cells

**Figure 1:**
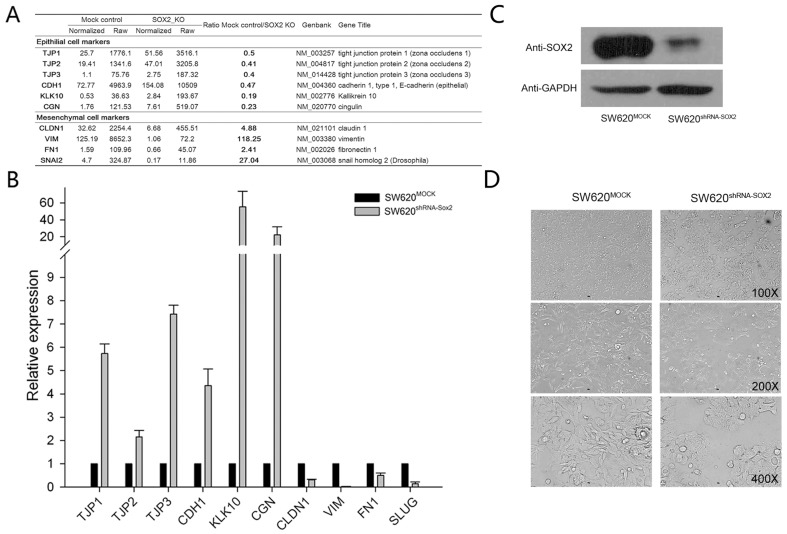
The expression of key genes related to the EMT process and the cell morphology changed in response to SOX2 knock down. (A) A microarray analysis of the expression changes of genes related to epithelial and mesenchymal cells between SOX2 knock down and mock knock down SW620 colorectal cancer cells. (B) Quantitative real time PCR validation of expression changes in (A). GAPDH was used for normalization. The data are reported as mean ± SEM. (C) Western blot analysis of the expression of SOX2 in SW620-mock and SW620-shRNA-SOX2 cells. (D) Cell morphology changes between SW620-mock cells and SW620-shRNA-SOX2 cells. The SW620-mock cells maintained a spindle and fibroblastoid morphology while the SW620-shRNA-SOX2 cell showed an epithelioid spreading morphology. Magnifications: the top panel, 100×; the middle panel, 200×; the bottom panel: 400×.

The epithelial to mesenchymal transition (EMT) process is marked by changed in the expression of epithelial and mesenchymal markers. In a previous study, we successfully knocked down SOX2 in a colorectal cancer cell line SW620, in which SOX2 is highly expressed [20]. We found that a number of epithelial markers including TJP1, TJP2, TJP3, CGN and KLK10 [5] were up-regulated in response to SOX2 knock down compared to mock control cells using microarrays [20]. In addition, a series of mesenchymal marker genes including VIM, FN1, SNAI2, CLDN1 [5] were down regulated in the SOX2 knock down cells compared with MOCK controls (Figure 1, A, B). Quantitative real-time PCR (with primers provided in table S1) confirmed the observed changes of the array data (Figure 1, A, C, B). These observations made us to hypothesize that SOX2 is involved in the EMT process. We then repeated the experiment (Figure 1C) and found that the colorectal cancer cell with the MOCK control (SW620-MOCK) cells maintained mesenchymal cell morphology with spindle and fibroblastoid shape cells, while the cells with SOX2 knock down (SW620-shRNA-SOX2) showed epithelial cell morphology with epithelioid spreading cells (Figure 1D). These changes are typical changes during the MET process [3], [5].

**Figure 2:**
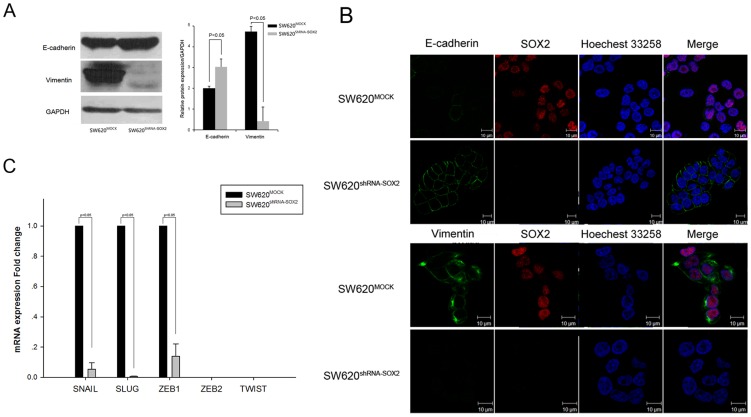
Silencing SOX2 induced a MET process in colorectal cancer cells. (A) Western blot analysis of expression of E-cadherin and vimentin in SW620-mock and SW620-shRNA-SOX2 cells. GAPDH was used as a loading control. The right panel showed the right panel quantified by the Quantity One software (Bio-Rad). GAPDH was used for normalization. (B) Immunofluorescence images of SW620-mock and SW620-shRNA-SOX2 cells stained with antibodies against E-cadherin, vimentin and SOX2. Secondary antibody was conjugated with FITC and Cy3. Cell nuclei were stained with Hoechest 33258. (C) Semi-quantitative RT-PCR analysis of EMT-related regulatory factors. GAPDH was used as a loading control.

We then assessed the expression of classical genes involved in the EMT process including vimentin (a mesenchymal marker), E-cadherin and ß-catenin [5] by western blot and immunofluorescence. We found that the expression of vimentin was almost completely abolished while the expression of E-cadherin was elevated significantly when SOX2 was knocked down (Figure 2A). Cell staining with SOX2 antibody confirmed the expression changes in E-cadherin and vimentin (Figure 2B). These results suggested that SOX2 is involved in the EMT process in colorectal cancer cells.

We next examined, by RT-PCR, the mRNA level of the core EMT regulatory factors Snail, Slug, Twist, Zeb1 and Zeb2 [3] and found that the mRNA expression of Snail, Slug and Zeb1 were significantly downregulated in SW620^shRNA-SOX2^ cells compared with MOCK cells (Figure 2C). The expression of Zeb2 and Twist were not detectable in either cell lines (Figure 2C). Additionally, we analyzed the effect of SOX2 known down on the expression of other SOX2 and TCF family members using the microarray data that we generated previously [20], and found that SOX8 and TCF3 was also down regulated by about 5.6 and 2 fold respectively after SOX2 knock down compared with the mock control (Table S2). However, the expression level of TCF2 was up regulated by about 2.2 fold after SOX2 knock down comparing with the mock control (Table S2).

To investigate whether overexpression of SOX2 could reverse the mesenchymal-epithelial transition (MET) process produced by SOX2 silencing, we overexpressed SOX2 as a SOX2-GFP construct in a SOX2 negative SW480 CRC cells. We confirmed by Western blot analysis that we were able to overexpress the SOX2 gene in SW480 cells (Figure S1A). RT-PCR analysis of the two markers of the EMT process showed that the E-cadherin expression did not change significantly (Figure S1B), but the expression of vimentin, the marker for the mesenchymal cells, increased dramatically (Figure S1B). However, the increase expression of vimentin seemed not enough to produce the phenotype at the cellular level as we did not observe the corresponding morphological changes related to EMT (data not shown).

### Inhibition of SOX2 in SW620 Cells Leads to Reduction in ß-catenin/TCF/LEF Signaling

**Figure 3:**
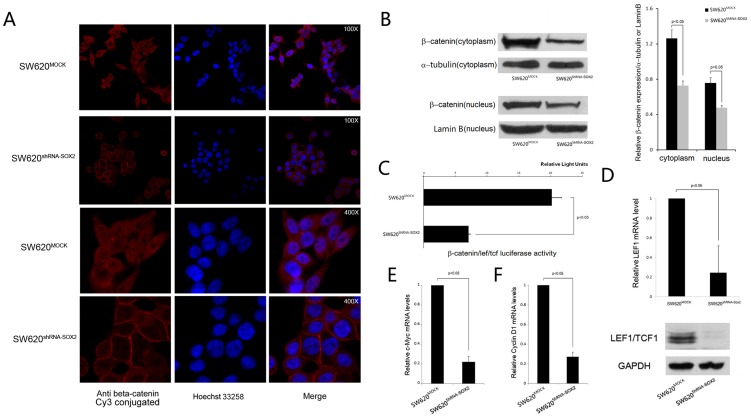
SOX2 is involved in the WNT signaling pathway in colorectal cancer cells. (A) Immunofluorescence images of SW620-mock and SW620-shRNA-SOX2 cells stained with anti-beta-catenin antibody. Secondary antibody was conjugated with CY3. Cell nuclei were stained with Hoechest 33258. The top 6 panels are in 100× magnification and the lower 6 panels are in 400× magnification. (B) Western blot analysis of beta-catenin expression using cytoplasmic and nuclear fractions of SW620-mock and SW620-shRNA-SOX2 cells. Alpha-tubulin and Lamin B were used as loading controls for the cytoplasmic and nuclear fractions respectively. The right panel showed the band intensities (left panel) quantified by the Quantity One software (Bio-Rad). (C) LEF1/TCF1-luciferase activity in SW620-mock and SW620-shRNA-SOX2 cells. (D) Quantitative real time PCR (upper panel) and western blot (lower panel) analyses of LEF1 in SW620-mock and SW620-shRNA-SOX2 cells. GAPDH was used as a loading control. (E) Quantitative real time PCR analysis of c-Myc gene in SW620-mock and SW620-shRNA-SOX2 cells. (F) Quantitative real time PCR analysis of cyclin D1 expression in SW620-mock and SW620-shRNA-SOX2 cells.

EMT is marked by nuclear translocation of ß-catenin and the activation of WNT/ß-catenin signaling pathway [21]–[24]. We stained SOX2 knock down and MOCK knock down colorectal cancer cells with ß-catenin antibody and found that there is an accumulation of ß-catenin in the cell membrane relative to cytoplasm and nucleus in the SOX2 knocked-down cells compared to the MOCK control (Figure 3A). A Western blot analysis further confirmed the reduced cytoplasmic and nuclear expression of ß-catenin in the SOX2 knocked-down cells compared to the MOCK control (Figure 3B). As upon translocation to the nucleus, ß-catenin serves as an activator of T-cell factor (TCF) and activates transcription of downstream targets in the canonical WNT pathway [25], we next measured the LEF/TCF activity using LEF/TCF reporter luciferase assay in the SOX2 knock down and MOCK knock down cells. We found that silencing SOX2 led to about 3-fold reduction of ß-catenin/TCF/LEF signaling activity (Figure 3C). Interestingly, the expression of TCF itself was also down regulated in the SOX2 knock down SW620 cells (Figure 3D), so were the expression of TCF specific target genes such as c-Myc and cyclin D1 (Figure 3E, F). Thus, we demonstrated that SOX2 knock down resulted in reduced ß-catenin nuclear translocation and reduced canonical WNT pathway activities.

### SOX2 affects cell migration/invasion via down regulation of MMP2 and its expression predicts liver metastasis and lymph node metastasis of CRC patients

**Figure 4:**
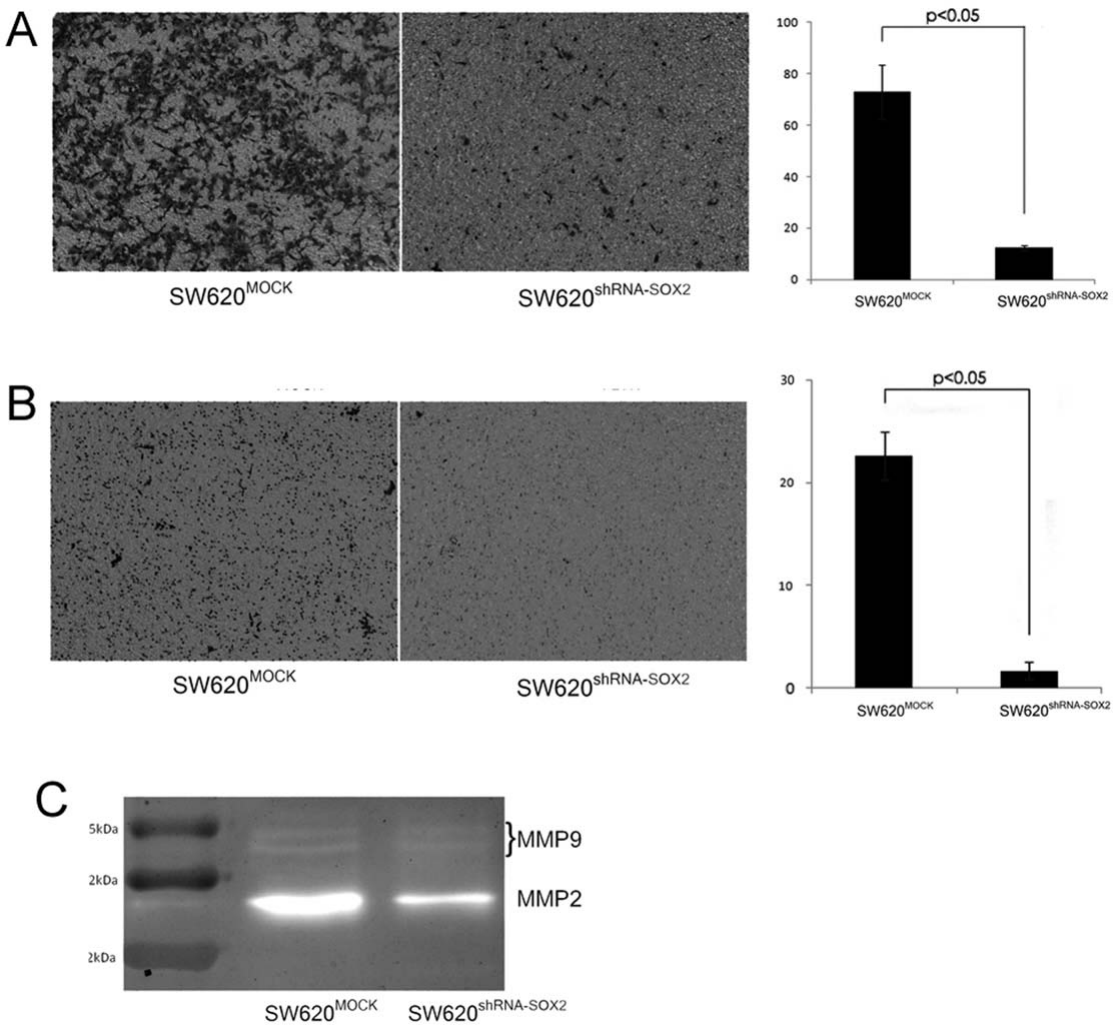
SOX2 is involved in cell migration and invasion in colorectal cancer cells. (A) Left panel, photographs of SW620-shRNA-SOX2 and SW620-mock cells using the Transwell migration assays; right panel, quantification data (mean ± SD) of the cells that migrated to the lower chambers. (B) Photographs (left panel) and the quantification results (mean ± SD) (right panel) of the cells that migrated to the lower chambers for SW620-shRNA-SOX2 and SW620-mock cells using the Transwell cell invasion assays. (C) Gelatin zymography assay for gelatinases (matrix metalloproteinases MMP2 and MMP9) in SW620-shRNA-SOX2 and SW620-mock cells.

We next assessed the SOX2's effect on cell migration and invasion using an established stable SW620 colorectal cancer cells expressing shRNA-SOX2 (SW620^shRNA-SOX2^) using transwell migration and invasion assays (Corning Inc., USA). We found that SOX2 knock down reduced the ability of cell migration by about 8 times (Figure 4A), and the ability of cell invasion by about 6 times (Figure 4B), compared with MOCK knock down cells. To understand if the gelatinases (matrix metalloproteinases MMP2 and MMP9) were involved in the SOX2 mediated invasion process, we measured MMP2 and MMP9 activities using gelatin zymography [26] and found that the activity of the MMP2, but not of the MMP9, was significantly decreased in SOX2 knock down cells comparing with MOCK knock down cells (Figure 4C).

**Figure 5:**
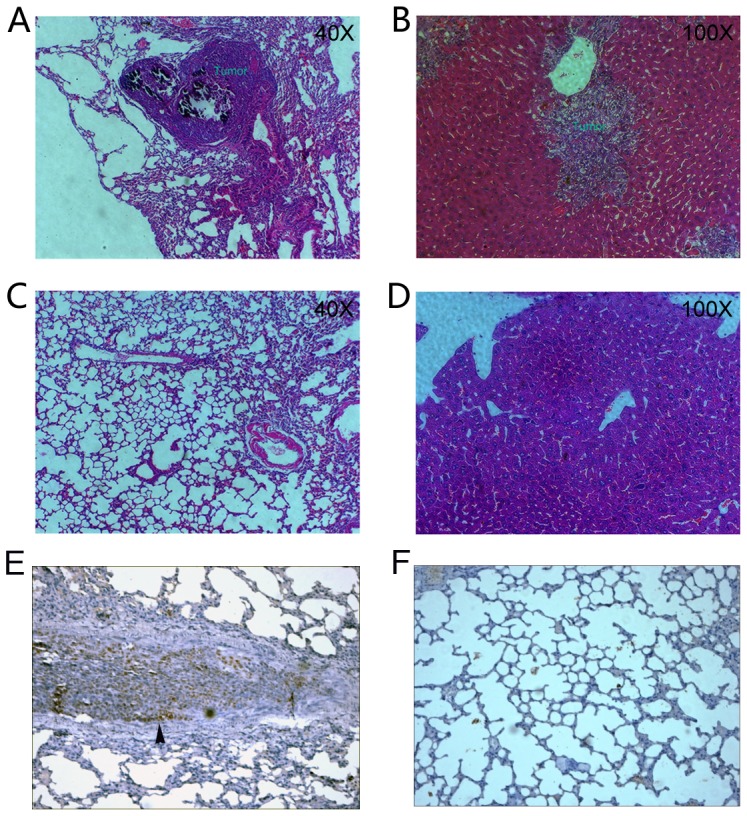
SOX2 knock down reduced *in vivo* metastasis of colorectal cancer cells in mice. (A) HE staining of mice lung tissue containing metastasis. (B) HE staining of mice liver tissue containing metastasis. (C) HE staining of mice lung tissue without metastasis. (D) HE staining of mice liver tissue without metastasis. (E) SOX2 staining of the lung metastasis with the arrow indicating strong staining of SOX2 in the invasive frontal regions. (F). SOX2 staining of the mouse lung tissue without metastasis. All images are in 100×.

After showing that knock down SOX2 reduced cell migration and invasion *in vitro*, we conducted an *in vivo* analysis. We compared the abilities of the SOX2 knock down and the mock knock down cells to form distant metastasis in mouse models. 5×10^6^ cells were injected respectively into the tail veins of 6-week-old female nude mice to introduce cells into the circulatory blood system. Mice were sacrificed on day 30 and lung and liver metastasis were examined using HE staining (Figure 5, A, B, C, D). 6 of 6 (100%) and 4 of 6 (60%) mice carrying the mock knock down cells had extensive lung and liver metastasis respectively. In contrast, only 3 of 7 (43%) and 2 of 7 (28.5%) mice injected with the SOX2 knock down cells had lung liver metastasis respectively. A statistical analysis (Chi-square test using log likelihood ratio) showed that the P values were 0.012 combing both lung and liver tumor metastatic events in mice. In addition, the numbers of tumor loci are about 6 times higher (3.3±1.75 vs. 0.57±0.79) in metastases with the mock knock down cells compared to the SOX2 knock down cells. These data suggested that SOX2 knock down reduced the ability of colorectal cancer cells to form metastasis.

**Figure 6:**
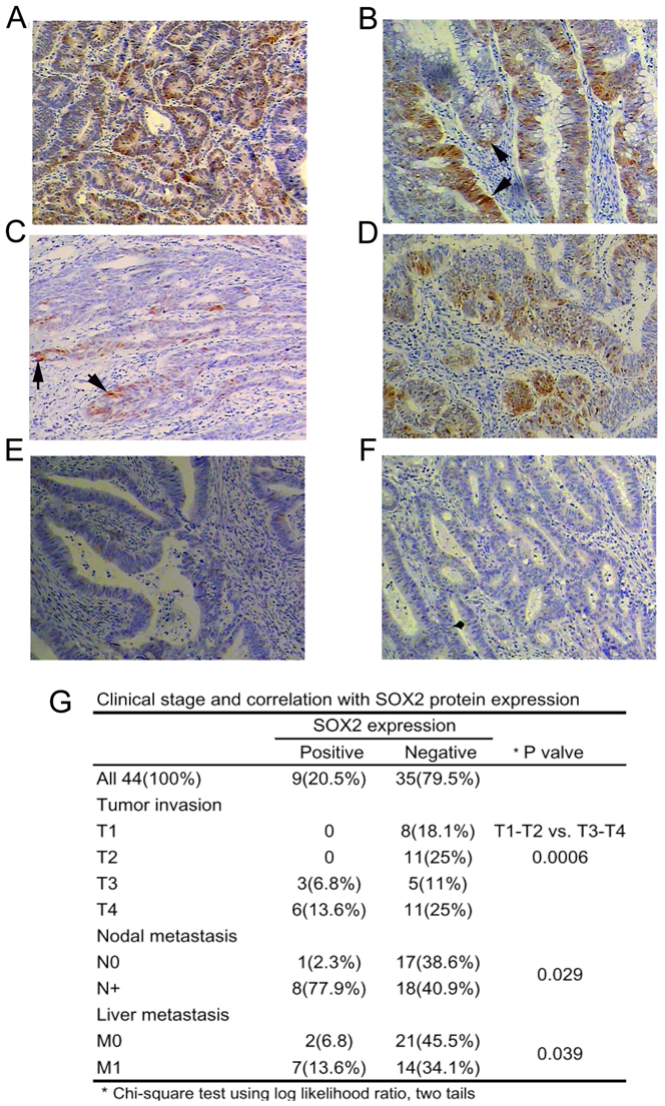
Immunohistochemical (IHC) staining of SOX2 in CRC tissues. (A–D) IHC images of SOX2-positve colorectal cancer tissues. Arrows indicate patches of strong SOX2 staining could indicate active tumor invasion fronts of the tumor capsules. (E–F) IHC images of SOX2-negative colorectal cancer tissues. All images are in 100×. (G) Statistical analysis of the relationship of SOX2 IHC staining with tumor stages, lymph node or liver metastases.

With the above *in intro* and *in vivo* data showing SOX2's role in invasion and metastasis in colorectal cancer cells, we asked the question whether SOX2 expression in primary human colorectal cancer tissues could predict tumor metastasis in human CRC patients. We examined SOX2 expression by IHC of paraffin-embedded tumor tissues from 44 CRC patients and found that ∼20% of patients (9/44) stained strongly with the SOX2 antibody (Figure 6A, B, C, D, E, F). In these SOX2 positive CRC tumors, the percentage of liver and lymph node metastasis were 77.8% and 88.8% (7/9 and 8/9 cases) respectively. However, in the SOX2 negative CRC tumors, the percentage of liver and lymph node metastasis were 40.0% and 51.4% (14/35 and 18/35 cases) respectively. The difference is statistically significant (P values of 0.029 and 0.039 respectively for lymph node and liver metastasis, Chi-square test using log likelihood ratio, two tails) (Figure 6G). We did not find any correlation between the SOX2 staining and the location of CRC to the right or left colon. However, we found that SOX2 staining correlated with advanced T3–T4 tumor stages, which are defined by tumor invasion through the muscularis propria into the pericolorectal tissues (T3) or by tumor penetration to the surface of the visceral peritoneum or to other organs or structures (T4) (T1–T2 tumors vs. T3–T4 tumors, P = 0.0006, Chi-square test using log likelihood ratio) (Figure 6G).

## Discussion

### SOX2's role in the Epithelial-Mesenchymal Transition (EMT) process in colorectal cancers

We demonstrated for the first time a connection between SOX2 and the Epithelial-Mesenchymal Transition (EMT) process [its reciprocal process is the Mesenchymal-Epithelial Transition (MET) process] [27]. During EMT, cells undergo morphological changes from the epithelial polarized morphology to the mesenchymal fibroblastoid morphology [27], [28]. After EMT process, epithelial cells lose cell-cell or cell-substrate contacts, and gain increased migratory capabilities. EMT is often marked by the loss of cell-cell adhesion molecules (e.g. E-cadherin), down-regulation of epithelial differentiation markers including cytokeratins and E-cadherin [27], [28]. In the meanwhile, mesenchymal markers such as vimentin, fibronectin and N-cadherin are up regulated [27], [28]. Our data on the morphological and molecular changes between SOX2 knock down and mock control cells reflected the corresponding MET process (the reversal process of the EMT process) (Figure 2), suggesting SOX2 is involved in the EMT process.

### SOX2's role in the Epithelial-Mesenchymal Transition (EMT) is mediated through the WNT pathway

The EMT is also marked by nuclear relocalization of ß-catenin [29]. We also observed that relocalization of ß-catenin from cytoplasmic/nuclear to membrane after SOX2 knock down. ß-catenin could form complexes in different cellular compartments including the adhesion complex at the plasma membrane and the signaling complex in the nucleus [30] (Figure 2A–B). Our data suggests a model for SOX2's role regulating the balance between ß-catenin in the adhesion complex at the plasma membrane and in the signaling complex in the nucleus EMT process: SOX2 could reduce the expression of E-cadherin at plasma membranes, thus reducing the binding capacity of the adhesion complex for ß-catenin. In the meantime, the WNT signaling components in the nucleus recruit more ß-catenin, activates WNT signaling and its downstream targets. The WNT pathway is widely regarded as the crucial pathway for colorectal carcinogenesis [31]–[33]. Our data also suggested that SOX2 activates WNT signaling activity as demonstrated by reduced LEF/TCF activity and reduced expression of well-known downstream target gene cyclin-D1 and c-Myc in SOX2-knock down cells (Figure 3). Our data in CRC is consistent with the report by Chen et al. who showed that SOX2 physically interacts with ß-catenin in human breast cancer cells [34]. The relationship between other SOX family proteins and ß-catenin has also been reported. For example, Zorn et al. showed that in Xenopus, two SOX family members (XSox17 alpha and beta, and XSox3) physically bind to beta-catenin and inhibit WNT pathway activity [35]. Zhang et al. showed that in Xenopus, XSox3 did not physically interact with beta-catenin, but could regulate Xnr5, a beta-catenin/VegT-regulated early zygotic gene, thereby indirectly involved in the WNT signaling [36].

The existence of a link between EMT and SOX2 is not surprising, as EMT is key developmental program in embryo development [37] and SOX2 plays a critical role in the early embryonic development and formation of tissues and organs [6]. Furthermore, the EMT process is often activated during cancer invasion and metastasis [37] and SOX2 is also over expressed in many types of cancers [15], [20], [34], [38], [39].

With multiple attempts, we over-express SOX2 (initially as SOX2-GFP construct, and later as SOX2 construct alone) in the previously SOX2 knock down CRC cells, and showed that we achieved SOX2 overexpression in the SOX2 knock down cells comparing to mock control (Data not show). However, we were not able to recue or reverse the MET process, or initiate an EMT process. We also overexpressed the SOX2 in the SOX2-negative SW480 CRC cells (Fig. S1A) and showed that vimentin expression increased dramatically (Figure S1B). However, the increase expression of vimentin did not generate the corresponding morphological changes related to EMT (data not shown). This observation, although to our surprise, might suggest that SOX2 interacts or works with other factors in the process. The discrepancy between the effect of gene knock down and over expression have prior incidences. For example, Burbridge *et al.* knock down of Dcdc2 resulted in hippocampal malformations and a bimodal migration pattern of the transfected neurons [40]. However, they showed that the treatment of shRNA-transfected neurons with the DCDC2 overexpression construct failed to rescue the migration phenotype [40]. Retinoid X receptor alpha-null (RXRalpha-null) mutants exhibit hypoplasia of their ventricular myocardium and die at the fetal stage [41]. However, Subbarayan et al. showed that RXRα overexpression in cardiomyocytes causes dilated cardiomyopathy but fails to rescue myocardial hypoplasia in RXRα-null fetuses [41]. Another example involves perlecan (Pln), which is a major heparan sulfate proteoglycan (HSPG) of extracellular matrices [42]. Savore et al. showed that addition of exogenous Pln does not rescue growth responses to FGF-2 in Pln knockdown C4-2B prostate cancer cells [42].

We showed that SOX2 silencing increased the expression of E-cadherin (Figure 2A). However, the regulating mechanism remains unknown. Snail and Slug are both transcriptional factors found to bind to the promoter regions of E-cadherin and repress E-cadherin expression during the EMT process [43]–[45]. In our analysis, we found that Snail, Slug and Zeb1 were down regulated after SOX2 silencing induced MET process. This suggested that SOX2 might induce the EMT process by acting through Snail, Slug or Zeb1. However, whether SOX2 directly or indirectly modulate these regulatory factors remains to be investigated.

### Knock down SOX2 reduced cell migration *in vitro* and invasion in mouse models and its expression predicts liver metastasis and lymph node metastasis of CRC patients

We further demonstrated that silencing SOX2 could significant decrease the ability of cell migration and invasion *in vitro* (Figure 4A–B) and reduce the ability of tumor metastasis *in vivo* (Figure 5). Matrix metalloproteinases (MMPs) are involved in tumor metastasis including tumor-induced angiogenesis, tumor invasion, and establishment of metastatic foci at the secondary site [46]. We showed by a gelatin zymography assay that the MMP2 activity was down regulated after SOX2 knocked down (Figure 4C), suggesting it is one of the mediators for the SOX2 effect on cell invasion/migration, and tumor metastasis. MMP2 has been shown to be important for metastasis in colorectal cancer [47], [48].

Finally, we showed that SOX2 expression correlated to more advanced tumor stages of T3–T4 (Fig. 6G). In addition, we also found that SOX2 expression in primary human colorectal cancer tissues could predict tumor metastasis in human CRC patients. However, we do not know whether SOX2 are expressed in the metastasis tissues because we did not have metastasis tissues for IHC staining. A supporting evidence for a role of SOX2 in metastasis is that we found that the primary cell line SW480 did not express SOX2 but the metastatic cell line SW620 expressed SOX2 (data not shown).

The SW480 and SW620 tumor cell lines are respective primary and metastatic colon adenocarcinoma cell lines established from the same patient [49].

In summary, we demonstrated that silencing SOX2 could induce a mesenchymal-epithelial transition in colorectal cancer cells, which partially explain the observation that SOX2 knock down lowered cell mobility and invasion ability in vitro and reduce metastasis *in vivo*. The molecular events involved in SOX2 silencing mediated phenotypic changes include expected changes in epithelial and mesenchymal markers during EMT, relocation of ß-catenin and reduced WNT pathway activity. However, the fact that we were not able to rescue, reverse or initiate the EMT process after overexpressing SOX2 (either alone or as SOX2-GFP) in a SW620 SOX2 KO background, suggesting that SOX2, although implicated in the maintenance of the EMT phenotype, is not an activator or initiator of the EMT process, or that SOX2 needs to work with other cofactors in this process. Finally, our data suggested that SOX2 could be used as a marker to predict liver/lymph node metastasis in colorectal cancer patients.

## Supporting Information

Figure S1 **Western blot analysis of SOX2 in the SOX2-negative SW480 CRC cells transfected with the SOX2-GFP or the GFP vectors (A), and RT-PCR analysis of the expression of vimentin and E-cadherin in SW480 CRC cells transfected with the SOX2-GFP or the GFP vectors (B).** The Y-axis in B is relative expression level, and the standard deviation bars showed data from three replicate experiments.
(TIF)

Table S1 **Quantitative real-time PCR primers.**
(XLS)

Table S2 **Changes in gene expression of the SOX and TCF familiy proteins afer SOX2 knock down compared with the mock-control.**
(XLS)
