# Supplementary figures and images for: Silencing SOX2 Induced Mesenchymal-Epithelial Transition and Its Expression Predicts Liver and Lymph Node Metastasis of CRC Patients
Source: PLoS One. 2012 Aug 17;7(8):e41335. doi: 10.1371/journal.pone.0041335 (PMC3422347; doi:10.1371/journal.pone.0041335)

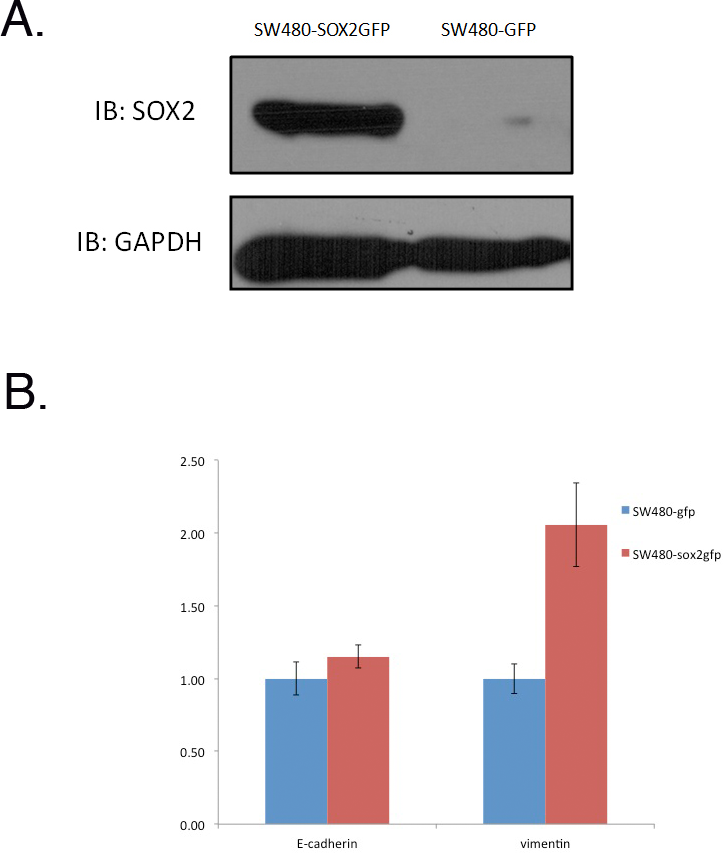

Supplement: Figure S1 — Western blot analysis of SOX2 in the SOX2-negative SW480 CRC cells transfected with the SOX2-GFP or the GFP vectors (A), and RT-PCR analysis of the expression of vimentin and E-cadherin in SW480 CRC cells transfected with the SOX2-GFP or the GFP vectors (B). The Y-axis in B is relative expression level, and the standard deviation bars showed data from three replicate experiments. (TIF) [file pone.0041335.s001.tif]
